# Therapeutic Perspectives in the Systemic Treatment of Kaposi’s Sarcoma

**DOI:** 10.3390/cancers14030484

**Published:** 2022-01-18

**Authors:** Marc-Antoine Valantin, Léna Royston, Maxime Hentzien, Aude Jary, Alain Makinson, Marianne Veyri, Sylvie Ronot-Bregigeon, Stéphane Isnard, Romain Palich, Jean-Pierre Routy

**Affiliations:** 1 Infectious Diseases Department, Pitié-Salpêtrière Hospital, AP-HP, Pierre Louis Epidemiology and Public Health Institute (iPLESP), INSERM U1136, Sorbonne University, 75013 Paris, France; romain.palich@aphp.fr; 2 Infectious Diseases and Immunity in Global Health Program & Chronic Viral Illness Service, McGill University Health Centre, Montréal, QC H4A3J1, Canada; stephane.isnard@mail.mcgill.ca (S.I.); jean-pierre.routy@mcgill.ca (J.-P.R.); 3 Division of Infectious Diseases, Geneva University Hospitals, 1205 Geneva, Switzerland; 4 Service de Médecine Interne, Maladies Infectieuses, Immunologie Clinique, CHU Robert Debré, 51090 Reims, France; mhentzien@chu-reims.fr; 5 Service de Virologie, Pitié-Salpêtrière Hospital, AP-HP, Pierre Louis Epidemiology and Public Health Institute (iPLESP), INSERM U1136, Sorbonne University, 75013 Paris, France; aude.jary@aphp.fr; 6 Infectious Diseases Department, INSERM U1175, University Hospital of Montpellier, 34000 Montpellier, France; a-makinson@chu-montpellier.fr; 7 Service d’Oncologie Médicale, Hôpitaux Universitaires Pitié Salpêtrière-Charles Foix, AP-HP, Pierre Louis Epidemiology and Public Health Institute (iPLESP), INSERM, Sorbonne University, 75013 Paris, France; marianne.veyri@aphp.fr; 8 Service d’Immuno-Hématologie Clinique, Hôpital Sainte-Marguerite, Aix Marseille Université, 13009 Marseille, France; sylvie.ronot@ap-hm.fr; 9 Division of Hematology, McGill University Health Centre, Montréal, QC H4A3J1, Canada

**Keywords:** Kaposi’s sarcoma, HIV, AIDS, antiretroviral, cancer

## Abstract

**Simple Summary:**

Alternative systemic treatments are needed for patients who develop chemotherapy-refractory KS. Anti-angiogenic therapies constitute interesting therapeutic targets in this context, due to the central role of angiogenesis in KS pathogenesis, and could represent attractive alternatives. Immune checkpoints blockade could also be an interesting therapeutic approach in order to restore anti-HHV-8 immunity and tumor control.

**Abstract:**

In patients with Kaposi’s sarcoma (KS), the therapeutic goal is to achieve a durable remission in the size and number of skin and visceral lesions. Although most patients show tumor regression in response to standard systemic chemotherapy regimens, alternative systemic treatments are needed for patients who develop refractory KS. Anti-angiogenic therapies represent attractive therapeutic targets in this context, due to the central role of angiogenesis in KS pathogenesis. Pomalidomide, which exhibits such anti-angiogenic activity through inhibition of VEGF, currently constitutes the most promising agent of this class and has been recently approved by the FDA. In addition, immune checkpoint blockade also represents an interesting alternative therapeutic approach through the restoration of immunity against HHV-8, the causative agent of KS, and improvement of tumor control. Although small series of cases treated successfully with these drugs have been reported, there is no marketing approval for anti-immune checkpoint antibodies for KS to date. In the present review, we will discuss potential therapeutic options for patients with recurrent or refractory KS, including systemic chemotherapies, immune checkpoint inhibitors, anti-herpesvirus agents, and anti-angiogenic drugs. Well-conducted clinical trials in this population are urgently needed to correctly address the efficacy of targeted agents and immunomodulators, while monitoring for adverse effects.

## 1. Introduction

Since 1996, combined antiretroviral therapy (cART) has dramatically decreased the risk of acquired immunodeficiency syndrome (AIDS), improved immune function and survival, and reduced the incidence of AIDS-defining cancers in people living with HIV (PLWH) [1,2,3]. Caused by human herpesvirus-8 (HHV-8), Kaposi’s sarcoma (KS) is an angio-proliferative malignancy with four distinct clinical forms depending on epidemiological and clinical criteria: epidemic or AIDS-related KS, classic KS in HIV-uninfected elderly individuals, endemic KS in HIV-uninfected individuals from Eastern/Central Africa, and iatrogenic/post-transplant KS. Kaposi sarcoma can also occur concomitantly with other HHV-8-associated diseases such as primary effusion lymphoma, multicentric Castleman disease, or KSHV-inflammatory cytokine syndrome (KICS).

In PLWH, cART initiation usually allows regression and prolonged stabilization of KS, especially mild forms of the disease; however, KS remains one of the most frequent tumors developing in PLWH, especially in men who have sex with men (MSM) [4,5]. In PLWH, the risk of developing KS remains thus significantly higher than in the general population, independently of cART initiation [6]. Moreover, recent reports suggest that a proportion of cART-treated PLWH with sustained HIV viral control and restored CD4 T cell counts develop a more limited form of KS, with restricted skin lesions that are usually less inflammatory than those observed among untreated PLWH [7,8,9]. The pathophysiology of this type of KS remains unclear and could be induced by immunosenescence, with an increased frequency of T cells exhibiting an immunosenescent phenotype (CD28^−^ and CD57^+^) in patients with KS compared to those without it [10].

The complexity of KS pathogenesis suggests multiple potential therapeutic targets. Alternative systemic treatments are needed, especially for patients with KS refractory to validated systemic chemotherapy regimens or exhibiting cardiotoxicity or peripheral neuropathy contraindicating anthracyclines or taxanes. Clinical trials are currently underway to assess the efficacy of anti-angiogenic drugs and immune checkpoints inhibitors in both HIV-positive and -negative patients with KS. In this review, we aim to discuss the current systemic approaches as well as new therapeutic targets for the treatment of KS.

## 2. Systemic Chemotherapy

As HHV-8 is not eradicable, patient with KS cannot be cured, and the goal of treatment is to achieve a durable remission of the skin or visceral lesions in size and number. The therapeutic management is based on an individual approach taking into account the criteria of disease extension, the localized or disseminated character of the lesions, predictors of disease evolution, in particular the immunovirological status of the patients, and the patient’s comorbidities [11].

Current therapeutic options include local and systemic treatments as chemotherapies and immunomodulatory drugs. In a retrospective study by Benajiba et al. analyzing the types of treatment received by patients with endemic or classical KS, local treatment was the most frequent (45%), followed by systemic therapies (41%, including doxorubicin, taxanes, and interferon), and therapeutic abstinence (14%) [12]. The choice of systemic treatment was influenced by the localization and clinical form of KS, the visceral involvement, and the time between first symptoms and diagnosis.

In patients with forms of KS in which immunosuppression is potentially reversible, adaptation of treatments constitutes the preferred option, such as modification of immunosuppressive therapies for post-transplant KS or optimal control of HIV infection with cART for AIDS-associated KS [13,14].

Systemic chemotherapy for KS is, however, warranted in patients with advanced or rapidly progressive disease and is currently based on liposomal anthracyclines and taxanes. Pegylated liposomal doxorubicin (PLD) and paclitaxel (PLX) constitute the preferred systemic chemotherapy regimens, PLD as first-line treatment at a dose of 20 mg/m^2^ every 3 weeks, and PLX as an alternative option at a dose of 100 mg/m^2^ every 14 days, with neutropenia as the most frequent dose-limiting toxicity [15]. The superiority of PLD over the combination of Adriamycin–vinblastine and bleomycin (AVB) has been demonstrated in a study by Northfeld et al. in 1998 [16]. The anti-tumor activity and safety of taxanes have also been evaluated, mostly in patients with classic KS and AIDS-associated KS, with an overall response of the cutaneous lesions ranging from 40 to 65% [17,18,19,20]. The concomitant use of systemic chemotherapy and cART with either PLD or PLX achieved a 60% response rate in a comparative study by Cianforca et al. No difference between cART + PLX (100 mg/m^2^ every 2 weeks) versus cART + PLD (20 mg/m^2^ every 3 weeks) was reported using an intention-to-treat analysis, but a trend toward increased Grade 3 to Grade 5 toxicity in the PLX arm (84% vs. 66%; *p*-value = 0.077) was observed [21].

Nab-paclitaxel and docetaxel have also been tested in small studies. A phase II trial with paclitaxel was conducted in six patients with advanced-stage skin KS, showing a response in all patients [22]. In a study by Fardet et al., nine and three patients with non-HIV advanced-stage KS received docetaxel and paclitaxel, respectively, with partial or complete response of KS lesions in all patients after chemotherapy [23]. Moreover, with docetaxel, a major response was obtained in visceral KS lesions, with the complete disappearance of digestive or respiratory symptoms. In another study evaluating docetaxel administered at a dose of 25 mg/m^2^ IV over 15–30 min weekly for 8 weeks to 12 patients with AIDS-associated KS previously treated with local or systemic agents, a partial cutaneous response in 5 patients was observed [24].

Other treatment options for subsequent lines of systemic chemotherapy, such as vinca alkaloids, bleomycin, and gemcitabine, can also be considered for AIDS- and non-AIDS-associated KS but are not recommended as first-line therapies [25,26,27,28].

Etoposide is the only oral systemic chemotherapy that demonstrated significant activity in KS [27,29]. In a recent study conducted in immunocompromised untreated PLWH, Krown et al. showed the superiority of PLD compared to a combination of bleomycin and vincristine, widely used in low- and middle-income countries, as well as compared to orally available etoposide, all combined with cART [30]. According to overall response and progression-free survival (PFS) rates at week 48, PLX + cART was superior on both investigational arms: PLX + cART was 30% more efficient than etoposide + cART (50%, 32 to 67; *n* = 59 vs. 20%, 6 to 33; *n* = 59) and 20% more efficient than bleomycin + vincristine + cART (64%, 55 to 73; *n* = 138 vs. 44%, 35 to 53; *n* = 132). In this study, etoposide was given orally as one 50 mg capsule twice per day on days 1–7 of each 21-day cycle, with escalation as tolerated on subsequent cycles up to a maximum of 100 mg taken twice per day.

Although essential in the management of HIV-related KS, cART alone is less effective than in combination with systemic chemotherapy in these patients. Reports show that partial and complete response ranged between 20% and 39% in patients receiving cART alone, with a statistical difference in favor of systemic chemotherapy + cART [31,32]. In addition, the choice of cART needs to be adapted to the type of systemic chemotherapy used. Indeed, the risk of drug–drug interactions between taxanes, protease inhibitors, and some non-nucleoside reverse-transcriptase inhibitors must be taken into account. Protease inhibitors (PIs), through their inhibitory effect on CYP3A4, induce a risk of chemotherapy toxicity; on the other hand, some non-nucleoside reverse-transcriptase inhibitors such efavirenz, nevirapine, and etravirine, through their inducing effect on CYP3A4, can reduce chemotherapy efficacy [33]. Paclitaxel is primarily metabolized by CYP2C8 and to a lesser extent by CYP3A4. Dolutegravir and rilpivirine are not expected to significantly impact CYP enzymes at clinically relevant concentrations. In addition, in vitro data suggest that PLX activates PXR, and such coadministration could potentially decrease dolutegravir and rilpivirine concentrations via induction of UGT1A1 for dolutegravir and via induction of CYP3A4 for rilpivirine, inducing potential virological failure [34]. Caution should thus be exercised when treating patients with PLX and dolutegravir and/or rilpivirine as concomitant therapy.

## 3. Anti-Herpesvirus Drugs in KS Treatment and Prevention

Kaposi sarcoma-associated herpesvirus (KSHV), also known as HHV-8, is the etiologic agent of all forms of KS. After primary infection, KSHV turns into a latency state, leading to the expression of a restrictive number of proteins: LANA-1 (latency-associated nuclear antigen), v-cyclin (viral cyclin), v-FLIP (viral FLICE inhibitory protein), kaposin, and various encoded microRNAs that promote, among others, cell growth and division, the inhibition of apoptosis, angiogenesis, and immune escape [35,36]. In KS, although most HHV-8-infected cells harbor viruses in a latent state, a little proportion of spindle cells (<5%) undergo lytic replication and produce new virions potentially involved in the proliferation of neighboring cells, suggesting their crucial roles in the process of tumorigenesis [37,38]. During this lytic cycle, HHV-8 expresses genes following a temporal and sequential expression pattern divided in three phases: immediate early (IE), early (E), and late (L) [39]. During the E phase, expressed early genes encode viral proteins primarily required for DNA replication and gene expression. Among them, the viral thymidine kinase (TK) encoded by ORF21 and the viral DNA polymerase encoded by ORF9 constitute targets of currently available anti-herpesvirus drugs. In KS, those two viral proteins may thus represent interesting therapeutic targets for treatment or prevention by inhibiting HHV-8 replication [40]. HHV-8 replication is essential during the early stage and persistence of HHV-8-associated diseases, including of KS [41,42,43,44]. The control of viral replication could then alleviate patients’ symptoms, improve disease course, and potentially avoid relapses especially in patients receiving immunosuppressive therapies [43,44]. Since there is no in vitro model of KS, the evaluation of potential antiviral drugs on HHV-8 replication has been mainly performed on primary effusion lymphoma (PEL) cell lines upon stimulation. In such models, ganciclovir (GCV), cidofovir (CDV), and foscarnet (Fos), as well as the thymidine analog North-methanocarbathymidine, have been reported to inhibit the production of KSHV, whereas acyclovir (ACV) did not exhibit any activity [45,46,47,48]. Furthermore, GCV, CDV, Fos, and ACV did not inhibit episomal virus DNA synthesis, confirming the lack of interest in these drugs in managing HHV-8 latent form and suggesting that episomal DNA is rather replicated by host DNA polymerase [49]. The HHV-8 TK and the HHV-8 phosphotransferase (PT, homologue to the UL97 protein kinase encoded by human cytomegalovirus) encoded by ORF36 were reported to induce GCV phosphorylation leading to cell death, with PT being more active than TK [50]. Other studies, however, demonstrated that HHV-8 TK efficiently phosphorylated zidovudine but not GCV, whereas KSHV PT preferentially phosphorylated purine analogues, leaving unexplained how GCV is activated into GCV triphosphate during HHV-8 infection [51,52]. In contrast, zidovudine (AZT) and stavudine competitively inhibited TK but not GCV and ACV [53]. More recently, Beauclair et al. explored the potential effects on KS of several tyrosine kinase inhibitors (TKI) approved by the FDA for other indications [54]. They could show that some of the tested TKI potently inhibited not only HHV-8 TK function but also viral lytic reactivation and the development of HHV8-infected endothelial tumors in mice. Although in vitro evidence supports a potential role of anti-herpesvirus drugs in the management of KS, the small amount of replicative KSHV in this disease suggests that a combination of these drugs with an activator of the lytic cycle or with agents targeting cellular processes and latent genes would be required [41,42].

The clinical efficacy of antivirals in KS and HHV-8-associated diseases has been only tested in case reports or small series, generally in combination with initial chemotherapy [43,55]. The efficacy of (val)GCV or Fos on HHV-8-viral load has been suggested by the clinical improvement induced by (val)GCV or CDV or Fos, or by the maintenance of remission achieved with (val)GCV [35,43,55,56,57,58,59]. In a randomized cross-over clinical trial testing a daily dose of 900 mg of valganciclovir versus placebo, Casper et al. found a significant reduction in the frequency and quantity of HHV-8 replication in 26 HHV-8-infected men [44]. However, conflicting results exist regarding the clinical efficacy of anti-herpetic agents in the treatment of KS [35,60,61,62]. One pilot clinical trial tested (val)GCV alone in five HIV-negative patients with classic KS and another one tested CDV alone in five HIV-positive and two HIV-negative patients with KS; in both studies, no clinical response could be observed [61,62].

Several studies have evaluated the risk of developing KS in PLWH [59,60,63,64]. In an English cohort started before the cART era, 3688 PLWH were included, among whom 568 developed KS. A significantly decreased adjusted risk for developing KS was found in patients who had received Fos [relative hazard (RH) = 0.38, *p* = 0.038] and GCV (RH = 0.39; *p* = 0.015), but not ACV (RH = 1.10; *p* = 0.40) [65].

Systemic symptoms frequently occur during KS, similarly to other HHV-8-related diseases such as Castleman’s disease, KSHV inflammatory cytokine syndrome (KICS), or IRIS–KS [66,67]. Only few treatments have been used for the treatment of KS-associated systemic symptoms. (Val)GCV or (val)GCV and high-dose zidovudine [68,69] have also exhibited clinical efficacy. One currently unpublished randomized clinical trial testing 4 weeks of (val)GCV before starting cART versus starting cART immediately in 38 patients (19 in each group) with disseminated KS and without associated Castleman’s disease showed significant reduction of the occurrence of severe IRIS–KS events (2 events in the (val)GCV vs. 12 events in the control group, adjusted incidence rate ratio = 0.10, *p* = 0.001) [70]. Moreover, in a recent publication, tocilizumab was associated with (val)GCV and high-dose zidovudine in three patients suffering from Castleman’s disease with a sustained clinical response [71].

## 4. Anti-Angiogenic Therapies in KS Treatment

Tumor-induced angiogenesis is crucial in the development of many cancers to ensure sufficient nutrient and oxygen supply and allow metastatic spread. Multiple proteins have been identified as angiogenic factors and are currently targeted by therapeutic agents. Illustrating the quest for efficient anti-angiogenic drugs, a basking shark cartilage extract was commercialized in the early 2000s (Neovastat or AE-941) based on the assumption that sharks are not affected by cancers [72]. Despite promising preclinical studies, phase III clinical trials did not confirm any benefit [73,74,75].

Aside from this particular setback, anti-angiogenic drugs are increasingly used in cancer clinical management. The effect of these drugs remains modest in both preclinical and clinical trials, with frequent intrinsic or acquired tumor resistance [76]. However, in addition to a direct role of angiogenesis on tumor expansion, mounting evidence indicates a crosstalk between vasculature and immunity. Angiogenesis is now considered a driver of immunosuppression and immune evasion in the tumor microenvironment [77], with vascular endothelial growth factor (VEGF), a key mediator of angiogenesis, reported to upregulate immune checkpoints leading to T cell exhaustion [78,79]. Combinations of anti-angiogenic drugs and immunotherapies appear thus promising and are already approved for renal cell carcinoma, non-small cell lung cancer, hepatocellular carcinoma, and endometrial carcinoma [77].

The link between angiogenesis and immune dysregulation is epitomized by the natural history of KS. Abundant vasculature with increased permeability constitutes an early and prominent histological characteristic of KS, forming purplish and reddish macules, papules, and nodules on the skin and other organs. Aberrant angiogenesis in KS results from the production of pro-angiogenic factors by HHV-8-infected spindle cells, including VEGF-A, platelet-derived growth factor (PDGF), and basic fibroblast growth factor (bFGF) [80,81,82]. These growth factors further stimulate capillary growth into the tumor, which increases oxygen and nutriment supply and ultimately allows tumor cell proliferation. Known HHV-8 viral proteins responsible for angiogenic mediators’ upregulation include viral IL-6 (vIL-6), latency-associated nuclear antigen (LANA), viral G protein-coupled receptor (vGPCR), and viral macrophage inflammatory protein (v-MIP) [83,84]. Thus, targeting angiogenesis in KS emerges as a new research priority.

In recent years, the most interesting results of anti-angiogenic drugs have been achieved with thalidomide and its derivatives (Table 1). After the tragic teratogenic side effects described since the late 1950s, thalidomide regained interest due to its powerful anti-angiogenic and immunomodulatory effects and has been primary used in multiple myeloma treatment. The thalidomide analogues pomalidomide and lenalidomide, which are better tolerated, also exhibit anti-angiogenesis capacities through inhibition of VEGF production and VEGF-induced PI3K–Akt pathway [85]. Such family of drugs seems appealing for the treatment of KS. In an open-labelled single-arm phase I/II trial, pomalidomide was tested in 18 patients with AIDS-related and 10 patients with classical KS, 75% of whom had already received chemotherapy [86]. Most patients presented with advanced cutaneous diseases, with exclusion of patients with visceral or pulmonary KS. Complete or partial response was reached in 67% after 12.5 months and in 80% after 10.5 months of HIV-positive and HIV-negative patients, respectively. Based on those results, the FDA approved in May 2020 pomalidomide for KS treatment in both HIV-positive (after failure of ART introduction) and HIV-negative patients [86]. Further larger trials are currently underway to assess the efficacy of pomalidomide for HIV-associated KS in sub-Saharan Africa (NCT03601806), in HIV-positive and -negative patients with KS, alone (NCT04577755), or in combination with liposomal doxorubicin (NCT02659930). Regarding lenalidomide, less favorable results were reported, with absence or up to 40% of partial response, depending on the evaluation criteria [87].

Targeting directly VEGF, bevacizumab is a humanized anti-VEGF-A monoclonal antibody that is licensed for various cancers, including colorectal, lung, and breast cancers. In 2012, a phase II study evaluated the effect of this drug in 17 PLWH with KS persistence or progression despite cART introduction and reported a 31% complete or partial response at 8 months [88]. However, another randomized study on 14 patients failed to show a significant effect of intralesional bevacizumab in upper airways KS lesions [89]. Another single-chain anti-VEGF-A antibody fragment, brolucizumab, approved for the treatment of neovascular age-related macular degeneration, was tested in a mice model of KS with significant tumor regression [96]. The inhibition of VEGF receptors (VEGFR-1 and VEGFR-2) has also been attempted with different molecules, including pazopanib, which inhibits angiogenesis through the inhibition of multiple receptor tyrosine kinases including VEGFR1 and VEGFR2 and is approved for renal cell carcinoma and soft tissue sarcoma treatment. Its off-label use has been reported to date only in a single patient with refractory classical KS, with complete response [90].

Given the importance during KS development of PDGF and c-KIT receptors, which are two receptor tyrosine kinases selectively inhibited by imatinib, the clinical utility of imatinib has also been tested in KS. In a pilot phase II study, 30 patients with HIV-related KS (with or without ART exposure) were treated with imatinib for a median duration of 22.5 weeks [91]. Ten patients (33.3%) achieved partial response, whereas 5 patients discontinued therapy due to adverse events. Sorafenib, another kinase inhibitor with activity against VEGF, c-kit, and PDGF receptors was also tested in a phase Ib study including 10 patients with HIV-related and classic KS. Sorafenib was, however, poorly tolerated, with 7/9 patients exhibiting toxicities possibly due to drug–drug interactions with ritonavir, and the response rate was only 29% [92].

Inhibitors of the mTOR pathway as sirolimus (rapamycin) and temsirolimus have also been reported to have activity on KS. In vitro, those agents were shown to exhibit anti-angiogenic properties by inhibiting VEGF secretion by KS tumor cells [97]. In solid organ transplant recipients, studies suggest that switching the immunosuppressive regimen from cyclosporin A to sirolimus results in KS regression [11,98]. This strategy, in addition to tapering down the immunosuppression, has now become the standard management of post-transplant KS [99]. However, in immunocompetent patients, the immunosuppressive properties of mTOR inhibitors seem to overcome its antitumor effect, with for instance no effect of everolimus reported in patients with classic KS [93]. Regarding KS in PLWH already receiving ART, Krown et al. reported that out of seven participants receiving sirolimus, three patients had a partial response, without increase of HIV viral loads [94]. Notably, all three patients were on PI-containing regimens, which induced considerable pharmacokinetic interactions and resulted in more than 200-fold differences in cumulative rapamycin doses compared to patients on NNRTI-containing regimens [94]. Altogether, even if mTOR inhibitors in KS are recommended for post-transplant KS, their use in other populations remains poorly attractive due to an unfavorable risk/benefit ratio.

## 5. Effects of HIV Protease Inhibitors on KS

Influencing several cellular pathways, HIV PIs have also been suggested to exert a direct inhibitory effect on AIDS-KS, independently of virological suppression and CD4 reconstitution [100]. Although still debated, this effect is believed to result in part from the anti-angiogenic properties of PIs, motivating their use in HIV-uninfected individuals with KS. In a clinical trial, 16 out of 26 HIV-uninfected patients with KS treated with indinavir were reported to improve or stabilize their lesions at 12 months [95]. A favorable clinical course was interestingly associated with higher indinavir plasmatic levels, reduced levels of bFGF, and decreased levels of HHV-8 antibody titers [95]. Nelfinavir, another PI, has also been reported to inhibit HHV-8 replication in vitro [101]. A phase II clinical trial is currently assessing the efficacy of nelfinavir in PLWH already on ART with KS (NCT03077451).

## 6. Immunotherapies in KS Treatment

Immunotherapy has dramatically improved the prognosis of many cancers, such as non-small-cell lung cancer, gastric carcinoma, head and neck squamous cell carcinoma, and renal cell carcinoma. These therapies consist in reversing the anergy of immune cells, induced by the tumor and its microenvironment, in order to efficiently kill tumor cells. Such a restoration of immune function is based on the blockade of immune checkpoints by monoclonal antibodies. Ipilimumab, blocking CTLA-4, was the first agent to be commercialized. It was followed by nivolumab and pembrolizumab, blocking PD-1, and finally by atezolizumab, targeting PD-L1.

Immunotherapy could constitute an interesting therapeutic approach in patients with KS [102,103,104]. On the one hand, the lack of specific anti-HHV-8 cellular immunity has been shown to participate in the occurrence of KS [105]. Blocking immune checkpoints could restore anti-HHV-8 immunity and help control the tumor process. On the other hand, PD-1 and PD-L1 expression within the tumor tissue, which has been shown to be strongly correlated with the efficacy of immunotherapy [106], has been reported in KS biopsies [107,108,109,110,111,112]. Of note, other biomarkers, such as tumor-infiltrating lymphocytes, mutational burden, or immune gene signatures, may be associated with the response to therapy and have not been studied in KS [106].

To date, there is no marketing approval for anti-immune checkpoint antibodies in KS. However, a few clinical cases and series, although including a limited number of patients, have been published (Table 2) [113,114,115,116,117,118,119,120,121]. These cases included mostly KS in HIV-negative people (endemic and classic KS), with a majority of patients having refractory diseases, and one study (nine patients) included AIDS-related KS. Most had received cytotoxic chemotherapies, the most common being anthracyclines and taxanes, as recommended by international guidelines. Other molecules, including vincristine or bleomycin, had also been used. Only few patients had received targeted therapy or angiogenesis inhibitors. These heavy therapeutic histories underline the potential role of immunotherapy as a salvage therapy in patients progressing despite receiving several lines of treatment. Almost all patients had skin involvement, and many of them had lymph node, visceral, and soft tissue extensions, sometimes with devastating muscle and bone damages. Immunotherapy, after 3 to 6 months of treatment, had a positive effect in a majority of cases, with approximately 60% of partial responses, 10% of complete remissions, and 30% of stable diseases, although these numbers should be taken with caution as they resulted from cases series and case reports and could be hampered by selection bias (Table 2).

The adverse event profile of immune checkpoint inhibitors has been evaluated in patients with cancer [123,124]. In general, immunotherapy is well tolerated, and adverse events are only rarely the cause of treatment interruption. Common treatment-related adverse events include fatigue, rash, diarrhea, pruritus, decreased appetite, and nausea. Grade 3 or 4 treatment-related adverse events are typically observed in 10–15% of patients, whereas drug-related serious adverse events occur in approximately 10% of patients. Immune-related adverse events are commonly mild to moderate, including skin, musculoskeletal, gastrointestinal, and endocrine impairments. However, some rare and serious side effects can occur with a real risk of death, including immune-induced pneumonia or cardiomyopathy. The management of immune-mediated adverse effects is mostly based on corticosteroid initiation [125]. Corticosteroids are, however, known to worsen KS clinical course, which could constitute an issue for patient receiving immunotherapy in this context. Elsewhere, several cohorts have shown that immunotherapy is well tolerated in PLWH [122,125,126,127,128], without immunological or virological effects in these patients. Anti-PD-1 may even have a beneficial effect on reducing the DNA HIV reservoir [129]. Finally, the risk of immune checkpoint inhibitors in KS might be influenced by the concomitant occurrence of other HHV-8-related diseases. This has been highlighted in a study by Uldrick et al., evaluating the safety of pembrolizumab in PLWH and cancer, which included six patients with KS [122]. In this study, one patient with a history of KICS developed an HHV-8-related B cell lymphoproliferation following pembrolizumab administration and died. The evaluation of pembrolizumab as a first-line treatment for AIDS-related KS is currently ongoing.

Apart from immune checkpoint inhibitors, interferon-α is also a biologic response modifier that has been shown to exert a direct antiviral effect against HHV-8 in KS-infected cells [130,131]. In the early days of the AIDS epidemic, interferon-α was evaluated as a treatment for AIDS-related KS, with only partial response, probably related to its antiproliferative effect rather than to immunomodulation [132]. In a more recent study, 10 patients received peg-interferon-α2a (180 micrograms/week) during 1 and 12 months. Clinical response to treatment was observed in nine patients, with a median PFS of 645 days, including seven patients with complete clinical response and one patient with partial response [133].

## 7. Conclusions

New therapeutic targets are needed to efficiently treat patients with chemotherapy-refractory KS. Anti-angiogenic therapies constitute potential therapeutical targets due to the central role of angiogenesis in KS pathogenesis and could represent alternatives to chemotherapy. Pomalidomide seems to currently constitute the most promising agent and has been recently approved by the FDA, while other pathways could be targeted, and innovative molecules are being tested. Combinations with other anticancer drugs, in particular, immunotherapies could enhance their efficacy and thus need further investigations. Although suggested by in vitro studies, the role of anti-herpetic agents in KS treatment remains unclear. It needs to be noted that only few studies were performed in sub-Saharan Africa, despite the disproportionate health burden caused by KS in this region. This knowledge gap may be explained by the unavailability of such expensive and novel therapies in low-income countries and highlights the critical need for research at a global level.

All in all, those drugs could, however, constitute elements of combination therapies for refractory KS, for instance combined with chemotherapy regimens, at least in high-income countries where those drugs are available. Immune checkpoints blockade also needs to be further evaluated in this context. The frequency of refractory KS as well as recently reported cases of relapsing AIDS-related KS despite suppressed HIV viremia and apparently restored immunity on cART highlight the need of such alternative treatments. However, larger well-conducted clinical trials will be necessary to shed light on the efficacy of immunotherapy, anti-angiogenic agents, and antiviral drugs in KS.

## Figures and Tables

**Table 1:** Clinical cases and series evaluating anti-angiogenic drugs for classic and AIDS-associated KS.

Study	Study Design	KS Type	Cases (*n*)	Previous Treatments	Clinical Presentation	Anti-Angiogenic Drug	Outcomes
Ramaswami [86], 2021	Open-label single-arm phase I/II	AIDS-KS Classic KS	18 10	Chemotherapy (75% of patients)	Advanced cutaneous KS, exclusion of patients with visceral involvement	Pomalidomide	AIDS-KS: Complete/partial response in 67% of patients at 12.5 months Classic KS: Complete/partial response in 80% of patients at 10.5 months
Pourcher [87], 2018	Open-label, single-arm, phase II	AIDS-KS	12	Chemotherapy	Cutaneous KS, one patient with visceral involvement	Lenalidomide	Complete/partial response in 40% of patients at 48 weeks
Uldrick [88], 2012	Open-label, single-arm, phase II	AIDS-KS	17	Chemotherapy (76%) Immunotherapy (65%) Radiation (29%)	Advanced cutaneous KS, exclusion of patients with visceral involvement	Bevacizumab	Complete/partial response in 31% of patients at 8 months
Ablanedo-Terrazas [89], 2014	Randomized, open-label, phase II	AIDS-KS	14: 7 treated/ 7 controls	-	KS lesions of the upper airway in the T0 stage	Intralesional bevacizumab	Complete/partial response in 43% of patients at 14 weeks, no difference between treated and control groups
Harris [90], 2018	Case report	Classic KS	1	Chemotherapy Radiation	Cutaneous KS	Brolucizumab	Complete regression of all lesions at 6 months
Koon [91], 2013	Open-label, single-arm, phase II	AIDS-KS	30	Chemotherapy (57%)	Cutaneous KS, exclusion of patients with visceral involvement	Imatinib	Partial response in 33% of patients, median time to response was 21 weeks
Uldrick [92], 2017	Open-label, single-arm, phase Ib	AIDS-KSClassic KS	10	Chemotherapy	Any stage	Sorafenib	Partial response in 3/7 patients (28%), poorly tolerated
Mourah [93], 2015	Open-label, single-arm, phase II	Classic KS	11	Chemotherapy (64%)	Cutaneous KS	Everolimus	Only 1 patient with partial response, progression in 8/11 patients
Krown [94], 2012	Open-label, single-arm, phase II	AIDS-KS	7	Chemotherapy	Cutaneous KS	Sirolimus	Partial response in 3/7 patients
Monini [95], 2016	Open-label, single-arm, phase II	Classic KS	28	Chemotherapy	Cutaneous KS	Indinavir	Complete remission in 1/26 patient, partial regression in 2/26 patients, improved disease in 5/26 patients, stabilization of disease in 8/26 patients

KS: Kaposi’s sarcoma. cART: combined antiretroviral treatment. AIDS: acquired immunodeficiency syndrome.

**Table 2:** Clinical cases and series evaluating immune checkpoints inhibitors in classic and AIDS-associated KS.

Study	Type of KS	Cases (*n*)	Previous Treatments	Clinical Presentation	Immunotherapy	Outcomes
Saller [113] 2018	Classic KS	1	Chemotherapy	Soft tissues and lymph node involvement	Pembrolizumab	At week 30: partial response
Delyon [114], 2018	Endemic KS	2	Chemotherapy + radiotherapy	Cutaneous involvement + muscular, bone, and lymph node extension	Nivolumab	At month 6: 2 partial responses
Delyon [115], 2020 (abstract)	Classic KS (8 patients) and endemic KS (9 patients)	17	Chemotherapy (12 patients), no treatment (5 patients)	Cutaneous involvement ± lymph node extension (6 patients)	Pembrolizumab	A month 6: 10 partial responses, 2 complete remissions, and 4 stable diseases (1 treatment stopping for adverse event)
Tabata [116], 2020	Classical KS	1	Chemotherapy + radiotherapy + surgery	Cutaneous, gastric, and lung involvement + soft tissues extension	Nivolumab + ipilimumab	At month 3: complete remission
Galanina [117], 2018	AIDS-associated KS	9	Chemotherapy (4 patients), bortezomib (3 patients), lenalidomide (3 patients), no treatment (3 patients) + antiretroviral therapy	Cutaneous, gastric, and lymph node involvement	Nivolumab (8 patients) or pembrolizumab (1 patient)	At month 2–6: 5 partial responses, 1 complete remission, 3 stable diseases
Kraehnke [118], 2019	Classic KS	1	-	Lymph node involvement	Ipilimumab	At month 3: complete remission
Zer [119], 2019 (abstract)	Classical KS	13	Progressive disease despite >1 line of systemic chemotherapy	Measurable disease by PET/CT and/or physical exam	Nivolumab + ipilimumab	At month 6: 4 partial responses, 1 complete remission, 6 stable diseases (1 non evaluable patient)
Gambichler [120], 2020	Classic KS	1	Chemotherapy	Soft tissues and lymph node involvement	Pembrolizumab	At month 6: partial response
Cesmeci [121], 2021	Classical KS	1	-	Cutaneous, gastric, bone, and lymph node involvement	Nivolumab	At month 6: complete remission
Uldrick [122], 2019	AIDS-associated KS	30, 6 KS, 5 NHL,19 non-AIDS-defining cancers.	Chemotherapy		Pembrolizumab	Over 183 cycles: grade 1 or 2 (*n* = 22), and 20% (*n* = 6) grade 3, 1 death. Complete response (lung, 1 patient), partial response (NHL, 2 patients), stable disease for 24 weeks or more (KS, 2 patients), stable disease for less than 24 weeks (15 patients), and progressive disease (8 patients).

KS: Kaposi’s sarcoma. cART: combined antiretroviral treatment. AIDS: acquired immunodeficiency syndrome, NHL: non-Hodgkin lymphoma.
